# Immunosuppressive Pathways in Cutaneous Melanoma: Functional Integration Between PD-1 and CD73 and Therapeutic Implications

**DOI:** 10.3390/ph19060913

**Published:** 2026-06-09

**Authors:** Rayana Vilela Bertolucci, Bruna Klein, Camilla Casarin Pase, Vitória Capelli de Melo, Margarete Dulce Bagatini

**Affiliations:** 1 Medical School, Federal University of Fronteira Sul, Campus Chapecó, Chapecó 89815-899, Santa Catarina, Brazil; rayanavilela@gmail.com (R.V.B.); camillapase1@gmail.com (C.C.P.); vitoriacapelli@hotmail.com (V.C.d.M.); 2 Postgraduate Program in Biomedical Sciences, Federal University of Fronteira Sul, Campus Chapecó, Chapecó 89815-899, Santa Catarina, Brazil; brunaklein06@yahoo.com.br

**Keywords:** melanoma, tumor microenvironment, immunosuppression, purinergic signaling, immune checkpoint inhibitors, resistance to immunotherapy

## Abstract

**Background:** Cutaneous melanoma (CM) is a highly immunogenic malignant neoplasm. It features high mutational burden and intense lymphocytic infiltration, supporting the use of immunotherapies, especially inhibitors of the programmed cell death protein 1 (PD-1) checkpoint. Despite advances with anti-PD-1 therapies, such as nivolumab and pembrolizumab, many patients still experience resistance. This result highlights additional immunosuppressive mechanisms within the tumor microenvironment (TME) that limit T-lymphocyte-mediated responses. **Objectives:** The aim was to discuss the immunologic and metabolic bases of PD-1- and CD73-mediated pathways and evidence that CD73 inhibition can boost PD-1 inhibitor efficacy by acting on convergent immunosuppressive pathways. **Methods:** We conducted a narrative literature review focusing on tumor immunosuppression, purinergic signaling and checkpoint inhibitor-based immunotherapy. **Results:** The purinergic pathway, mediated by the ectonucleotidase CD73, is a critical regulator of tumor immunosuppression. CD73 converts extracellular adenosine monophosphate (AMP) into adenosine. This adenosine accumulates in the hypoxic and inflamed TME, exerting immunosuppressive effects. Adenosine acts as a “metabolic brake,” inhibiting proliferation, cytokine production, and cytotoxic activity of CD8^+^ T lymphocytes and natural killer (NK) cells. It also promotes the expansion of regulatory T cells (Tregs) and tumor progression. This axis may limit responses to PD-1 blockade, suggesting that complementary pathways are active. **Conclusions:** Integration of PD-1 and CD73 pathways suggests that CD73 inhibition may enhance PD-1 blockade by targeting convergent immunosuppressive mechanisms. This supports the exploration of combination strategies to broaden the benefits of immunotherapy in CM.

## 1. Introduction

Cutaneous melanoma is a malignant skin neoplasm characterized by abnormal proliferation of melanocytes in the epidermis, known for its high invasiveness and potential to metastasize [1,2]. Even though it comprises a relatively small percentage of skin cancer cases, melanoma remains the leading cause of skin cancer mortality, accounting for over 80% of related deaths [3,4]. Among the various risk factors, ultraviolet (UV) radiation plays a significant role in its development. Globally, CM is the ninth most common malignant neoplasm. Among skin cancers specifically, it has the second-highest mortality rate [5]. In 2020, there were an estimated 325,000 new melanoma cases worldwide, with projections indicating this could reach 500,000 to 600,000 cases by 2040 [5].

Melanocytes are pigmented cells that synthesize melanin. Melanin is a pigment that determines skin and hair color; it also possesses antioxidant properties and absorbs UV radiation [6,7]. In the skin, various stimuli, especially UV radiation, activate these cells. As a result, tyrosine is converted into melanin [6]. Individuals with lower melanin concentrations, such as those with fair skin, are more susceptible to developing melanoma [7], which occurs more frequently in sun-exposed areas [4].

Clinically, the disease is suspected when a pigmented, asymmetric lesion, which may exhibit irregular borders, color variation, or changes in shape and size, appears [4]. Early-stage diagnosis followed by surgical excision is associated with a favorable prognosis. In contrast, surgery alone is limited in cases of locally advanced or metastatic disease [8,9]. The five-year survival rate ranges from 69% to 99% for stage I, II, and III melanomas [10] but drops to about 35% with distant metastases [11].

Melanoma is characterized by its high immunogenicity, a trait that enables initial recognition of tumor cells by the immune system and contributes to disease control in early stages [5,12]. This occurs through the recognition of melanoma antigens by tumor-infiltrating lymphocytes (TILs), cells that mediate the antitumor immune response, whose presence correlates with improved survival and a lower risk of metastasis [9,12]. However, as the neoplasm progresses, immune evasion mechanisms begin to predominate within the TME, limiting the efficacy of antitumor responses [13]. Persistent antigenic stimulation and chronic inflammation favor functional exhaustion of effector immune cells, promoting tumor growth and metastatic dissemination [5].

In this context, immunotherapy has revolutionized the treatment of advanced melanoma, primarily by introducing immune checkpoint inhibitor (ICI) blockade [5,14]. Monoclonal antibodies targeting PD-1, such as nivolumab and pembrolizumab, have led to significant improvements in patient survival [2,15]. Although anti-PD-1 inhibitors have substantially improved melanoma treatment, a considerable proportion of patients still fail to respond to monotherapy, suggesting that additional immunosuppressive mechanisms within the TME may limit the efficacy of ICIs [15].

Metabolic alterations within the tumor environment also contribute to immune evasion and therapeutic resistance. Among these mechanisms, dysregulation of the adenosine pathway and increased CD73 activity have emerged as important mediators of tumor-associated immunosuppression [14,16]. Inhibition of CD73 has been associated with enhanced antitumor immune responses and reduced metastatic progression [16]. Accordingly, the combination of selective CD73 inhibitors with anti-PD-1 antibodies has emerged as a promising therapeutic strategy to improve treatment efficacy [14].

Given this interaction, this review examines the role of CD73 inhibition in enhancing the efficacy of PD-1 inhibitors in CM, along with preclinical and clinical evidence supporting this combination. Furthermore, it discusses whether this effect results from the modulation of convergent immunosuppressive pathways that limit T-lymphocyte activation.

## 2. Immunogenicity, Tumor Microenvironment and Immune Escape in Cutaneous Melanoma

Cutaneous melanoma is among the most immunogenic solid tumors in humans [17], mainly due to its high mutational capacity [18,19] and resulting generation of diverse tumor neoantigens [20]. Chronic UV radiation exposure induces specific mutations, causing genomic changes [21,22] that promote initial immune recognition by adaptive immune cells, especially CD8^+^ T lymphocytes [23,24].

This high immunogenicity sets melanoma apart from other tumors by enabling stronger immune recognition and response, which helps explain the clinical success of immune checkpoint blockade therapies [25,26], especially those targeting the PD-1/PD-L1 axis [27,28]. High tumor mutational burden (TMB) is directly associated with the production of neoantigens presented by major histocompatibility complex (MHC) molecules [29], increasing the likelihood of recognition by T-cell receptors [30]. Several studies demonstrate a positive correlation between high TMB, T-lymphocyte infiltration, and improved responses to immunotherapies [31,32], positioning melanoma as a paradigmatic model for studying the interaction between tumor immunogenicity and therapeutic efficacy. However, this same genomic instability contributes to intratumoral heterogeneity, thereby favoring adaptive immune-escape mechanisms during disease progression [33].

The tumor microenvironment of CM is a highly dynamic and heterogeneous structure, in which tumor cells, immune populations, stromal components, and the extracellular matrix (ECM) interact to regulate immune evasion, tumor progression, and therapeutic responsiveness [34,35].

Within the immune compartment, the TME of melanoma shows varying levels of cytotoxic CD8^+^ T lymphocytes, CD4^+^ helper T lymphocytes [36], Tregs [17], myeloid-derived suppressor cells (MDSCs) [37], and tumor-associated macrophages (TAMs) [38]. CD8^+^ T lymphocytes represent the primary effectors of antitumor immunity [23,24], capable of recognizing and eliminating tumor cells by releasing granzymes and perforin B [39]. However, their functional activity is strongly modulated by the local immunological context and may be progressively suppressed by regulatory cells and inhibitory signals within the TME [33].

Tumor-infiltrating lymphocytes are important prognostic and predictive markers in CM [40,41]. High density of cytotoxic CD8^+^ T lymphocytes within the TME is associated with improved survival and better responses to PD-1 inhibitors [42,43]. By contrast, tumors with fewer effector T lymphocytes and dominant immunosuppressive circuits often show primary resistance to immunomodulatory therapies [43,44]. Moreover, the functional state of TILs matters; chronic exposure to tumor antigens can exhaust these cells [45,46], as evidenced by high expression of inhibitory receptors, such as PD-1, and reduced proliferative and cytotoxic capacity.

CD4^+^ T lymphocytes exert dual functions in melanoma, supporting cytotoxic responses [36,47] and promoting immunosuppression, particularly when differentiated into Tregs [47,48]. These regulatory cells frequently accumulate within the TME and are associated with the secretion of immunosuppressive cytokines, such as interleukin-10 (IL-10) and transforming growth factor beta (TGF-β) [49,50], as well as with elevated expression of ectonucleotidases involved in purinergic metabolism, such as CD39 and CD73 [12,51,52]. Complementarily, MDSCs and TAMs, particularly those polarized toward an M2-like phenotype, contribute to the inhibition of lymphocyte activation and ECM remodeling and to the promotion of tumor angiogenesis [41,53].

In addition to the cellular component, the ECM critically influences the architecture and function of the TME in melanoma. Changes in ECM composition and stiffness directly affect immune cell migration and the availability of soluble factors, and the transmission of mechanical and biochemical signals [54,55]. Additionally, excess collagen and other structural components can create physical barriers to lymphocyte infiltration, thereby promoting a microenvironment permissive to immune evasion [56].

Another central aspect of TME organization is the presence of tumor hypoxia. This condition results from uncontrolled tumor growth and dysfunctional vascularization [57]. Hypoxia activates transcription factors such as hypoxia-inducible factor-1α (HIF-1α). These factors drive metabolic and immunological alterations [58]. Notable changes include increased anaerobic glycolysis, microenvironmental acidification [59], and the induction of immunosuppressive molecules such as PD-L1 and the CD39/CD73/adenosine purinergic axis [60]. These adaptations restrict the availability of essential nutrients for T lymphocytes. As a result, lymphocyte proliferation and effector function are impaired [61].

The metabolic reprogramming of the melanoma TME, therefore, not only affects tumor cells but also imposes functional constraints on immune system cells [33,61,62]. Tumor and immune cells compete for glucose, amino acids, and oxygen, while lactate and adenosine build up, making the environment hostile to T lymphocytes [61,62]. In this context, metabolic and signaling pathways converge to establish a state of local immune tolerance that favors tumor progression and reduces the efficacy of therapies targeting immune checkpoints.

In summary, melanoma exhibits a complex immunologic dynamic in which pro-inflammatory signals coexist with robust mechanisms of local immunosuppression [24]. Although the initial immunogenicity favors activation of antitumor responses, tumor progression is frequently associated with the selection of clones that modulate the microenvironment to promote immune evasion [63]. The cellular composition, metabolic landscape and structural organization of the TME collectively promote T-cell dysfunction and therapeutic resistance.

This duality, high immunogenicity combined with efficient escape mechanisms, makes melanoma a particularly relevant setting for investigating convergent immunosuppressive pathways, such as those mediated by the PD-1 axis and CD73-dependent purinergic metabolism. Therefore, understanding these interactions between immune signaling, metabolic reprogramming and microenvironmental immunosuppression is essential to the development of combined therapeutic strategies capable of restoring effective antitumor immunity and improving the clinical efficacy of immunotherapy.

## 3. PD-1 Signaling in Cutaneous Melanoma: Functional Basis, Therapeutic Applications and Mechanisms of Resistance

The PD-1 receptor is a central immune checkpoint regulating lymphocyte activation and peripheral tolerance [64,65]. Structurally, PD-1 is a transmembrane protein in the immunoglobulin superfamily [66], predominantly expressed on activated T lymphocytes, but also present on B cells, NK cells, and myeloid subpopulations [67,68]. Its ligands, PD-L1 and PD-L2, have distinct expression patterns: PD-L1 is broadly expressed by tumor cells, stromal cells, and infiltrating immune cells [65,69], while PD-L2 is mainly in antigen-presenting cells [70]. PD-1 ligand binding recruits phosphatases, such as SHP-2, which dephosphorylate key components of the T-cell receptor (TCR) signaling cascade and co-stimulatory pathways, ultimately attenuating lymphocyte activation [71,72].

From a functional perspective, PD-1 signaling profoundly impacts T-lymphocyte biology in melanoma. Sustained activation of this checkpoint is associated with functional exhaustion, a state characterized by reduced proliferation [73], decreased production of pro-inflammatory cytokines such as tumor necrosis factor alpha (TNF-α) and interleukin-2 (IL-2) [64], and progressive loss of cytotoxic capacity [74]. In addition, PD-1 acts as a signaling brake proximal to the TCR, promotes the metabolic reprogramming of T cells, reduces activation-induced glycolysis and limits the energy availability required for sustained effector responses [75]. In the melanoma TME, characterized by hypoxia and intense metabolic competition, this reprogramming contributes to the functional impairment of infiltrating T cells [76].

The clinical importance of the PD-1/PD-L1 axis in melanoma became clear with the arrival of the monoclonal antibodies nivolumab and pembrolizumab. These treatments transformed care for advanced disease [77]. These agents can at least partially restore suppressed T-lymphocyte function, so the immune system can better recognize and remove tumor cells [78].

Despite these advances, many patients do not respond to PD-1 blockade. Others may develop resistance after initially responding, showing the limitations of this strategy [79]. Primary resistance can occur even in tumors with high mutational burden and strong lymphocytic infiltration. This means that the presence of antigen and T cells alone does not guarantee a clinical response [79]. In contrast, acquired resistance arises from tumor and microenvironment adaptations. These changes include alterations in antigen presentation, growth of immunosuppressive populations, and increased metabolic barriers [79].

In this context, it is increasingly evident that the efficacy of PD-1 inhibitors is modulated by additional factors within the TME. These include parallel metabolic and immunosuppressive mechanisms that may limit the sustained functional restoration of T lymphocytes.

## 4. CD73 as a Regulator of the Immunosuppressive Microenvironment in Cutaneous Melanoma

### 4.1. CD73 Biology and Adenosine Signaling

The cell-surface enzyme CD73 (ecto-5′-nucleotidase, NT5E), which is glycosylphosphatidylinositol (GPI)-anchored, is expressed in a variety of cells, including stromal and TME cells, immune cells, normal tissue cells, and tumor cells. Its primary function, involving the final step of the purinergic pathway, is the conversion of extracellular AMP into adenosine, which has anti-inflammatory and immunosuppressive effects. CD73 also regulates cell adhesion, signaling, and tumor progression [80,81].

**Figure 1:**
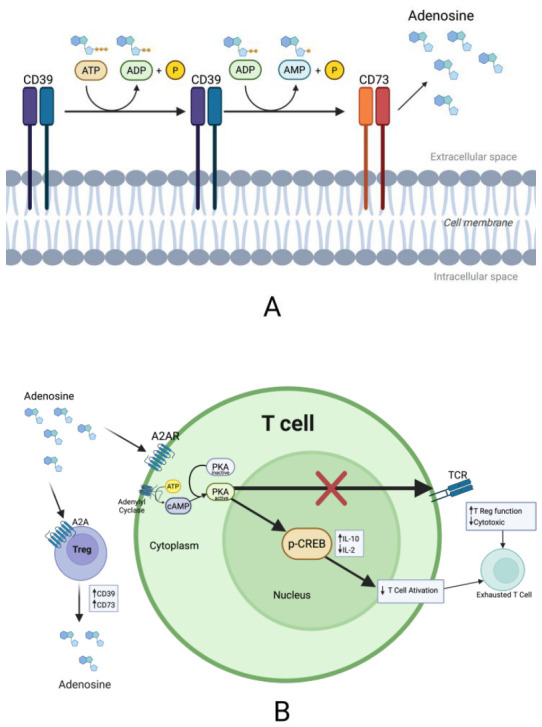
Adenosine formation in TME. (**A**) CD39 degrades extracellular ATP into ADP and AMP, which is converted into adenosine by the ectonucleotidase CD73. (**B**) The adenosine produced binds to the A2A receptor on T lymphocytes, promoting the activation of Adenylyl Cyclase and conversion of ATP into cAMP. The increase in cAMP activates protein kinase A (PKA), leading to phosphorylation of CREB in the nucleus, increased (↑) IL-10 production, and reduced (↓) IL-2 production, resulting in decreased (↓) T-lymphocyte activation. In addition, PKA inhibits (×) TCR signaling, reduces (↓) cytotoxicity, and favors the function of regulatory T cells (Tregs), thereby inducing a state of T-lymphocyte exhaustion. Additionally, adenosine stimulates Tregs, through CD39 and CD73 expression, to produce more adenosine, establishing a positive feedback mechanism. (**A**) Created in BioRender. Pase, C. (2026) https://BioRender.com/nfmjovz. (**B**) Created in BioRender. Pase, C. (2026) https://BioRender.com/p9vb813.

This activity integrates the CD39/CD73 enzymatic axis, a classical catabolic pathway that metabolizes extracellular nucleotides. Under cellular stress, inflammation, or tissue damage, Adenosine Triphosphate (ATP) is released into the extracellular milieu. CD39 then hydrolyzes ATP into Adenosine Diphosphate (ADP) and AMP. CD73 completes the process by converting AMP into adenosine [81] (Figure 1A). Through the CD39/CD73 axis, adenosine binds to one of its four receptors. This exerts regulatory functions that remodel the TME toward an immunosuppressive state, reduce antitumor immune responses, and favor immune evasion by tumor cells [82].

The regulatory functions of adenosine in the TME are mediated by four type 1 purinergic receptors, which are G protein-coupled: A1, A2A, A2B, and A3. These receptors differ in their affinity for adenosine, distribution, signaling properties, and functional receptor type [82,83]. A1 and A3 receptors are generally associated with Gi-dependent signaling pathways and may exert context-dependent effects on tumor-cell survival and apoptosis [83,84,85].

However, the immunosuppressive effects of adenosine in cancer are predominantly mediated by A2A and A2B receptors [82,83]. Widely expressed in immune system cells, such as myeloid cells and lymphocytes, these receptors activate Gs protein-dependent pathways, increasing intracellular cAMP and contributing to the suppression of the immune response [83].

The A2A receptor (A2AR) is present on CD4^+^ and CD8^+^ T cells, NK cells, dendritic cells (DCs), and macrophages. In the TME, hypoxia and inflammation promote adenosine accumulation and persistent A2AR activation. In immune cells, A2AR signaling increases intracellular cAMP and activates PKA, impairing signaling needed for TCR activation and pro-inflammatory gene transcription. Consequently, CD8^+^ T lymphocytes reduce proliferation, impair IL-2 production, decrease secretion of antitumor cytokines such as interferon-γ (IFN-γ) and tumor necrosis factor alpha (TNF-α) and decrease the cytotoxic activity of T cells and NK cells. These effects establish A2AR as a major negative regulator of antitumor immunity within the TME (Figure 1B) [86].

Beyond its direct effects on effector lymphocytes, adenosine signaling also promotes Treg expansion and function. Tregs express high levels of CD39 and CD73, generating additional adenosine and reinforcing a positive feedback loop that sustains local immunosuppression [87]. In tumor cells, A2AR signaling may additionally promote EMT and resistance to apoptosis through the Ras-associated protein 1 (Rap1) and PI3K/AKT pathways [86].

The A2B receptor, in turn, exhibits low affinity for adenosine and is primarily activated in environments with high adenosine concentrations, as observed in the inflammatory or hypoxic TME. Its activation is associated with the promotion of angiogenesis, increased tumor proliferation, induction of epithelial–mesenchymal transition (EMT), and facilitation of metastatic processes. Moreover, this receptor plays an important role in modulating cancer-associated fibroblasts and regulating the chronic inflammatory response, thereby contributing to tumor progression [47,88].

Experimental evidence indicates a positive feedback loop mediated by the adenosine-A2B-CD73 axis. Activation of the A2B receptor upregulates CD73 expression, increasing adenosine production and immunosuppressive signaling in the TME [89,90]. In this context, therapeutic strategies targeting signaling through A2A and A2B receptors have been investigated. CD73 inhibitors and adenosine receptor antagonists offer a promising way to restore immune antitumor activity and enhance the efficacy of cancer immunotherapy [82,83].

Collectively, these pathways establish the CD73–adenosine axis as a central regulator of immune suppression and tumor progression within the melanoma microenvironment.

### 4.2. Immunosuppressive and Protumoral Effects of CD73

Consistent with these mechanisms, increased CD73 expression has been associated with multiple protumoral and immunosuppressive effects across several malignancies. CD73 expression is increased in various types of cancer compared with corresponding normal tissues, including esophageal, head and neck, lung, and pancreatic carcinomas. In the majority of neoplasms, elevated levels of this ectonucleotidase are associated with poorer overall survival and with higher immune scores across various tumor types, suggesting its role in modulating the tumor immune microenvironment [91,92]. From a clinical perspective, elevated CD73 levels have also been associated with poorer prognosis and resistance to conventional therapies, such as chemotherapy and radiotherapy [93].

However, this association is not universal. In specific tumors, such as renal cell carcinoma and endometrial carcinoma, CD73 expression may be associated with a favorable prognosis [91,92]. These apparently contradictory findings suggest that the biological and prognostic impact of CD73 may be highly context-dependent. Factors such as tumor type, the immune composition of the TME, the relative contribution of stromal versus tumor-cell CD73 expression, and differences in purinergic signaling dynamics may influence whether CD73 predominantly exerts immunosuppressive or context-specific regulatory functions.

In tumor cells, high CD73 expression is directly associated with promoting cell proliferation, migration, and invasion, as well as facilitating angiogenesis and metastasis. This protumoral activity is largely mediated by adenosine signaling through A2A and A2B receptors on the cancer cells themselves and on neighboring cells, thereby creating a TME that is favorable to cancer growth [89,90,93].

Beyond tumor cells, CD73 is widely expressed in the TME by cancer-associated fibroblasts (CAFs), endothelial cells, and various immune cell populations. In CAFs, CD73 expression contributes to ECM remodeling and to the maintenance of an immunosuppressive environment. In addition, tumor-cell-derived exosomes may carry CD73 and CD39, thereby enhancing adenosine production in the TME and promoting disease progression [82,90,93,94].

Within the immune compartment, CD73 plays a central role in the negative regulation of the antitumor response, exhibiting elevated expression in Tregs, NK cells, TAMs, and DCs [92,95,96]. In these populations, CD73 promotes adenosine production and mediates multiple immunosuppressive effects. In Tregs, adenosine suppresses the activity of effector T cells. In NK cells, elevated CD73 levels reduce cytotoxicity and cytokine production. Adenosine also promotes the polarization of TAMs toward the M2 phenotype, which is characterized by immunosuppressive and protumoral properties. In dendritic cells, adenosine impairs maturation and antigen-presenting capacity, thereby compromising activation of adaptive immune response [92,95,96,97].

Taken together, these observations support the notion that CD73 contributes significantly to CM progression through both protumoral and immunosuppressive mechanisms. By simultaneously suppressing effector immune responses and promoting protumoral cellular phenotypes, CD73 establishes a permissive microenvironment for immune evasion and metastatic progression.

### 4.3. Prognostic Relevance of CD73 in Cutaneous Melanoma

In CM, CD73 has emerged as an important prognostic marker, primarily due to its direct involvement in tumor progression and in the modulation of the immune microenvironment. Increased expression of this ectonucleotidase is associated with more aggressive tumors and with effective mechanisms of evasion of the antitumor immune response. Thus, CD73 has been recognized as a mediator of the clinical course of the disease [98,99].

Several studies demonstrate an association between high levels of CD73 expression and unfavorable clinical outcomes. In patients with CM, elevated CD73 expression correlates with poor prognosis not only as a biomarker of aggressive disease but also because it reflects the establishment of a metabolically immunosuppressive TME enriched in adenosine signaling and dysfunctional antitumor immunity. Taken together, a significant reduction in progression-free survival is observed, as CD73 promotes fundamental processes involved in tumor dissemination, including cellular migration, tissue invasion, and the establishment of distant metastases. In this context, elevated CD73 levels are associated with metastases in lymph nodes and visceral organs, characterized by high invasive potential [98,99,100].

Another relevant aspect concerns CD73’s versatility as a biomarker. Its quantification in tumor tissue or in serum provides important information for risk stratification and clinical decision making [99,101,102]. Immunohistochemical analysis allows for direct identification of CD73 expression in the primary tumor and metastatic sites, enabling evaluation of the TME. In parallel, the detection of soluble CD73 in serum has emerged as a promising, minimally invasive alternative. Studies indicate that elevated serum levels of this enzyme before treatment initiation are independent predictors of mortality [99,101,103]. Nevertheless, important limitations remain regarding the clinical implementation of CD73 as a biomarker, particularly due to heterogeneity in detection approaches and differences in the evaluation of soluble and tissue-associated CD73.

The clinical relevance of CD73 has become even more evident with the consolidation of immunotherapies in advanced melanoma treatment. In the context of immune checkpoint blockade using antibodies such as anti-PD-1 and anti-CTLA-4, elevated CD73 expression has been described as a marker of therapeutic resistance. Patients with low CD73 expression tend to exhibit more favorable responses to immunotherapy, whereas those with high CD73 expression are often resistant to treatment [93,98,102].

The involvement of CD73 in multiple processes associated with tumor progression, immunosuppression, and therapeutic resistance establishes this enzyme as a promising target for pharmacological interventions [91,98]. In this context, CD73 blockade has been explored as a relevant therapeutic strategy to restore antitumor immunity, particularly in combination with ICIs, such as anti-PD-1 antibodies, with the aim of overcoming resistance mechanisms and enhancing the efficacy of immunotherapy [104,105].

## 5. PD-1–CD73 Axis in Cutaneous Melanoma: Functional Convergence in T-Lymphocyte Suppression and Therapeutic Implications

The progression of CM is closely associated with the establishment of an immunosuppressive TME that can restrict the activation, clonal expansion, and effector function of T lymphocytes [24]. However, immunosuppression in melanoma does not result from a single isolated pathway but rather from the functional interaction of multiple overlapping regulatory mechanisms that collectively sustain immune evasion and therapeutic resistance [106]. This concept is particularly relevant for understanding why highly immunogenic tumors may still exhibit primary resistance or loss of response to immunotherapy.

These resistance mechanisms span both tumor-intrinsic and TME-associated processes and can be classified as primary resistance, adaptive immune resistance, and acquired resistance [107]. Mutations in Janus kinases 1 and 2 (JAK1/2) and loss of beta-2 microglobulin (B2M) lead to defects in IFN-γ signaling and antigen presentation, thereby promoting tumor immune evasion [108]. Additionally, active WNT/β-catenin signaling in tumors is associated with T-cell exclusion within the TME, thereby preventing lymphocyte infiltration into the tumor [109]. However, even when T cells successfully infiltrate the tumor, several immunosuppressive mechanisms can inhibit their activity, including Tregs, indoleamine 2,3-dioxygenase (IDO), lymphocyte activation gene 3 (LAG-3), and T-cell immunoglobulin and mucin domain 3 (TIM-3) [110]. Among these pathways, metabolic immunosuppression mediated by the CD73/adenosine axis stands out, and its close interaction with the PD-1 pathway constitutes the focus of this article.

**Figure 2:**
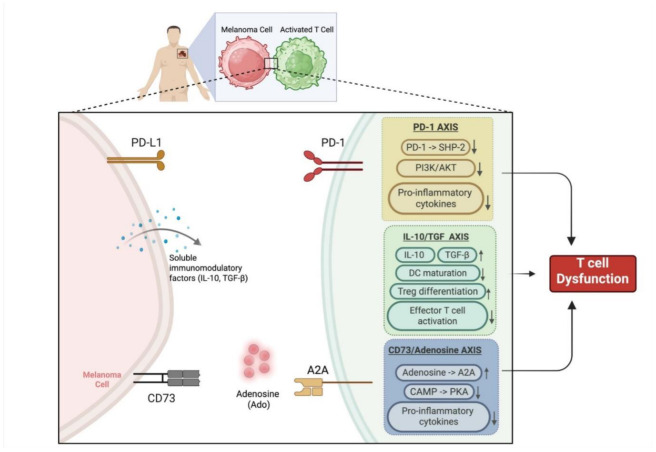
Schematic representation of the PD-1/CD73 axis and adenosine-mediated immunosuppression in the melanoma tumor microenvironment. Created in BioRender. Bagatini, M. (2026) https://BioRender.com/8kzj0du. The diagram highlights the coordinated immunosuppressive mechanisms driving T-cell dysfunction and tumor immune evasion through distinct molecular pathways. Engagement of the PD-1/PD-L1 axis triggers SHP-2–dependent inhibition of the PI3K/AKT pathway, reducing pro-inflammatory cytokine production. Concurrently, the CD73/Adenosine axis, driven by tumor-expressed CD73, leads to extracellular adenosine accumulation that signals through the A2A receptor on T cells, activating the immunosuppressive cAMP/PKA pathway. In parallel, soluble immunomodulatory factors such as melanoma-derived IL-10 and TGF-β limit dendritic cell maturation, promote Treg differentiation, and directly inhibit effector T-cell activation. Together, these pathways suppress TCR signaling, reduce pro-inflammatory cytokine production, sustain T-cell exhaustion, and limit effective antitumor immune response. Increased (↑); Decreased (↓).

These two pathways act as major interconnected regulators of immune dysfunction in melanoma. PD-1 signaling suppresses T-cell receptor activation through SHP-2 recruitment, reducing cytokine production, impairing cytotoxic activity, and promoting T-cell exhaustion [64,65,111]. While classical checkpoint pathways play a major role in immune regulation, immunosuppressive metabolism has also emerged as a central axis in regulating immunity within the TME [112]. In parallel, extracellular ATP is sequentially degraded by CD39 and CD73 into adenosine, which accumulates within the TME and exerts potent immunosuppressive effects through activation of A2A and A2B receptors on immune cells [113]. By acting on specific receptors present on T lymphocytes, NK cells, and antigen-presenting cells, adenosine suppresses pro-inflammatory cytokine production, reduces cytotoxicity, and promotes regulatory phenotypes (Figure 2) [87,114].

Although mediated by distinct molecular mechanisms, PD-1 signaling and the immune CD73 pathway immunosuppression functionally converge to impair T-lymphocyte activity within the TME. PD-1 activation inhibits proximal TCR signaling and contributes to metabolic reprogramming that reduces glycolytic activity and effector-cell fitness [115,116]. Simultaneously, adenosine signaling through A2A receptors increases intracellular cAMP levels, further suppressing T-cell activation, cytokine production, and cytotoxic function [113]. As a result, T lymphocytes remain present within the TME but become functionally exhausted and unable to sustain effective antitumor responses [72,115].

In melanoma, PD-L1 expression may also be induced by IFN-γ from infiltrating T lymphocytes, establishing a mechanism of adaptive immune resistance that links inflammation to suppression of cytotoxicity [117]. Together, these pathways establish a metabolically and immunologically suppressive microenvironment in which purinergic signaling and PD-1-mediated inhibition sustain T-cell exhaustion, favor immune tolerance, and limit sustained antitumor immunity.

In addition to checkpoint and purinergic signaling, melanoma-associated immunosuppression is further reinforced by soluble mediators such as IL-10 and TGF-β. These cytokines support Tregs differentiation, block dendritic cell maturation, and limit effector T-lymphocyte activation (Figure 2) [118]. Together with immune checkpoints and immunosuppressive metabolism, these soluble mediators amplify inhibition of the antitumor immune response.

The diagram highlights the coordinated immunosuppressive mechanisms driving T-cell dysfunction and tumor immune evasion through distinct molecular pathways. Engagement of the PD-1/PD-L1 axis triggers SHP-2-dependent inhibition of the PI3K/AKT pathway, reducing pro-inflammatory cytokine production. Concurrently, the CD73/adenosine axis, driven by tumor-expressed CD73, leads to extracellular adenosine accumulation that signals through the A2A receptor on T cells, activating the immunosuppressive cAMP/PKA pathway. In parallel, soluble immunomodulatory factors such as melanoma-derived IL-10 and TGF-β limit dendritic cell maturation, promote Treg differentiation, and directly inhibit effector T-cell activation. Together, these pathways suppress TCR signaling, reduce pro-inflammatory cytokine production, sustain T-cell exhaustion, and limit effective antitumor immune response.

Together, these pathways impose complementary metabolic and signaling restrictions that sustain lymphocyte exhaustion and local tolerance, even in the presence of antigen [113]. The efficacy of PD-1 inhibitors is modulated by factors related to the adenosine axis, as it imposes a basal metabolic brake on T-lymphocyte function [119]. Purinergic signaling through A2A and A2B receptors may persist even after PD-1 blockade, limiting the magnitude and durability of immune reactivation [120]. Moreover, PD-1 signaling may increase T-cell sensitivity to adenosine-mediated immunosuppression, establishing a negative feedback loop [121].

Consistent with this model, murine tumor studies demonstrate that PD-1 blockade increases A2A receptor expression in tumor-infiltrating CD8+ T cells, making these T cells more susceptible to adenosine-mediated suppression. As a result, A2A antagonists markedly enhance anti-PD-1 efficacy but have limited effects as monotherapy in certain contexts. Furthermore, anti-PD-1 treatment in both murine and human cancers is linked to upregulation of A2AR and CD73. Notably, resistance to PD-1/PD-L1 can be reversed by A2A antagonists, such as ciforadenant, in patients with renal cell carcinoma [121].

Therefore, the link between PD-1 signaling and the CD73/adenosine axis shows that tumor immunosuppression arises from interconnected metabolic and signaling pathways, not separate ones. This suggests that blocking CD73, by reducing purinergic suppression, could relieve TME pressure and improve the efficacy of anti-PD-1 immunotherapy in CM.

## 6. Preclinical, Translational, and Clinical Evidence for the Combination of Anti-PD-1 Therapy and CD73 Inhibitors

Growing evidence shows that the CD73–adenosine axis is a central immunosuppressive pathway in the tumor microenvironment of CM. It plays a major role in both primary and acquired resistance to PD-1 immune checkpoint inhibitors [14,99]. Importantly, evidence supporting the therapeutic targeting of this pathway originates from two distinct levels of investigation.

Preclinical evidence, derived predominantly from immunocompetent murine melanoma models, establishes the mechanistic rationale for dual blockade of PD-1 signaling and the adenosine pathway by demonstrating restoration of T-cell effector function, reversal of immunometabolic suppression, and enhanced responsiveness to checkpoint inhibition [93,98,122].

Clinical and translational evidence in humans, by contrast, remains primarily observational and biomarker-driven, linking CD73 expression and activity to resistance to anti-PD-1 therapy, adverse prognosis, and potential therapeutic vulnerability while providing early support for the clinical development of combination strategies targeting the CD73–adenosine axis [14,99,122].

### 6.1. Evidence Derived from Preclinical Murine Melanoma Models

Murine melanoma models have been essential to demonstrating that the CD73–adenosine axis is a key mechanism of immunosuppression in the TME. These models enable systematic assessment of combination therapies with PD-1 inhibitors [89]. Preclinical data show that CD73-mediated adenosine signaling acts as a metabolic brake on antitumor immunity in immunocompetent mice, making the TME resistant to immunotherapy [102,122]. Adenosine accumulation in the TME increases CD8^+^ T-cell exhaustion and reduces the effectiveness of anti-PD-1 monotherapy, providing a metabolic pathway for tumor escape [89].

Among these models, B16-F10, derived from a highly aggressive and poorly immunogenic murine melanoma, is a widely validated system for studying immune evasion and resistance to immunotherapy. Pioneering studies have demonstrated that genetic deletion or pharmacological inhibition of CD73 results in significant tumor control, associated with reduced generation of extracellular adenosine and the consequent reversal of local immune suppression, particularly when combined with PD-1/PD-L1 blockade or other ICIs (Table 1) [89,122,123]. Moreover, in these studies, the absence of CD73 in the tumor compartment led to decreased tumor growth and reduced pulmonary metastasis formation, highlighting its role in tumor progression and immune escape [124]. Despite their mechanistic relevance, caution is required when extrapolating preclinical efficacy data to clinical settings.

Studies with emerging pharmacological CD73 inhibitors have shown that effective inhibition of adenosine generation potentiates the antitumor activity of PD-1 blockade. This results in positive impacts on the frequency and function of tumor-infiltrating antitumor T lymphocytes. It also reduces mediators of cellular exhaustion and increases markers of cytotoxicity [91]. Preclinical studies have also shown that CD73 antagonists or selective inhibitors reduce adenosine concentrations in the TME. This promotes increased proliferation of CD8^+^ T lymphocytes, enhanced production of IFN-γ, and the production of other pro-inflammatory cytokines. In addition, there is a reduction in immunosuppressive phenotypes associated with regulatory T cells and MDSCs [89].

Consistent with these observations, recent preclinical investigations further strengthen the rationale for targeting the CD73–adenosine pathway in combination with checkpoint blockade. Experimental studies using selective CD73 inhibition, including quemliclustat (AB680), have demonstrated suppression of extracellular adenosine generation, restoration of T-cell effector activity, increased CD8^+^ T-cell infiltration, and improved responsiveness to PD-1/PD-L1 blockade across immunocompetent tumor models [14,89,122]. In melanoma and other solid tumors, these findings consistently indicate that CD73 blockade can reverse adenosine-mediated immunosuppression, favorably remodel the tumor microenvironment, and potentiate the efficacy of immune checkpoint inhibition, thereby supporting the biological rationale for combination immunotherapy [82,89,91,122].

Beyond CD73-targeted strategies, experimental studies have also explored the blockade of the adenosine A2A receptor as a complementary mechanism to disrupt adenosine-mediated immunosuppression in melanoma. In B16-F10 melanoma cells, treatment with istradefylline (IST), a selective antiparkinsonian A2AR antagonist, reduced cell viability and proliferation, potentiated paclitaxel-induced cytotoxicity, and modulated purinergic enzyme activity toward a profile less favorable to adenosine accumulation [125]. In a melanoma-bearing mice study, IST further induced a pro-inflammatory immune profile and inhibited AKT/mTOR signaling, a pathway critically involved in tumor growth and survival [125]. These findings suggest that selective A2AR antagonism may represent an effective strategy for disrupting the adenosine axis in melanoma, complementing CD73-targeted approaches.

### 6.2. Evidence Derived from Clinical and Translational Human Studies

In contrast to mechanistic evidence generated in preclinical murine models, evidence in humans is derived predominantly from translational biomarker studies, retrospective clinical cohorts, and early-phase therapeutic investigations in patients with cutaneous melanoma. Studies have shown that the expression and activity of the ectonucleotidase CD73 in the TME are linked to unfavorable clinical outcomes. CD73, which generates immunosuppressive adenosine, is associated with reduced responses to PD-1 inhibitor therapy and a poorer prognosis in advanced disease [16].

A retrospective multicenter cohort included 546 patients with advanced melanoma treated with anti-PD-1 therapy (nivolumab or pembrolizumab). Elevated soluble CD73 (sCD73) enzymatic activity in plasma, measured before treatment initiation, was significantly associated with lower clinical response rates and worse overall survival (OS) and progression-free survival (PFS). This association remained significant in multivariate analyses, even after adjusting for established clinical prognostic factors such as serum lactate dehydrogenase and presence of brain metastases. During treatment, sCD73 activity stayed stable and did not decrease with therapeutic response [99].

Similar findings were observed in smaller studies involving patients with metastatic melanoma treated with nivolumab. Elevated CD73 activity levels prior to therapy were associated with decreased overall survival and progression-free survival. These results indicate that baseline sCD73 measurement may facilitate the identification of subgroups with diminished clinical benefit from anti-PD-1 monotherapy [103].

The translational relevance of CD73 as a prognostic marker and potential mediator of resistance also appears in analyses of circulating immune subpopulations. In melanoma patients treated with nivolumab, a higher frequency of CD8^+^ T lymphocytes co-expressing CD73 and PD-1 in peripheral blood before treatment was associated with poorer overall survival. This suggests that co-expression of molecules involved in metabolic suppression and exhaustion could limit the efficacy of checkpoint therapy [15].

From the perspective of the TME, analyses of CD73 expression in human tumors have demonstrated that more than half of metastatic melanoma samples exhibit significant CD73 expression in tumor cells by immunohistochemistry, and in some series, this expression has been associated with poorer overall survival. However, the prognostic relevance of CD73 may vary depending on its cellular localization and distribution within the TME, as expression in tumor cells and in distinct immune infiltrate populations may exert distinct biological functions, requiring careful interpretation of the data [101].

Moreover, emerging evidence indicates that CD73 present in circulating exosomes may be involved in mechanisms of resistance to PD-1 inhibitor monotherapy in melanoma. In a retrospective pilot study, exosomes isolated from the plasma of patients treated with nivolumab or pembrolizumab exhibited functional CD73 activity, generating adenosine and reducing IFN-γ and granzyme B production by activated T-cells in vitro. In this context, patients who did not respond to therapy showed higher levels of exosome-associated CD73 during the early phases of treatment, suggesting a possible association between this pathway and a lack of clinical response [16].

These translational findings show that both tumor CD73 expression and sCD73 activity in blood are potential prognostic biomarkers. Both are associated with clinical resistance to anti-PD-1 therapy in CM. This reinforces the need for prospective studies of adenosine pathway modulation as a strategy to improve therapeutic responses.

Beyond their prognostic implications, these findings also provide a translational rationale for the therapeutic targeting of the CD73–adenosine axis in melanoma. Early-phase clinical trials evaluating anti-CD73 strategies, including oleclumab (MEDI9447), BMS-986179, and quemliclustat (AB680), in melanoma and other advanced solid tumors have demonstrated biological activity, pharmacodynamic modulation of adenosine signaling, and acceptable safety profiles, supporting further investigation of combination approaches with PD-1/PD-L1 blockade [120,126,127,128].

No predictive biomarker has yet been validated for this combination, although several candidates have emerged from translational studies. CD73 is among the most extensively studied: elevated baseline expression in tumor tissue and increased soluble CD73 (sCD73) in plasma have both been associated with poorer responses to anti-PD-1 therapy, supporting their potential predictive value [99,101,103]. Transcriptomic approaches have provided an additional layer of stratification. The adenosine signature reflects activity of the CD39/CD73/adenosine pathway and identifies tumors with high adenosinergic drive [129], whereas the innate anti-PD-1 resistance (IPRES) signature distinguishes tumors that are intrinsically refractory to PD-1 blockade [130]. Additional candidates include A2AR expression, the density of infiltrating CD8^+^ T cells, and the co-expression of CD73 and PD-1 on circulating T cells [15]. To date, however, all these candidates remain investigational and have yet to enter clinical practice.

Importantly, most currently available clinical evidence is retrospective, exploratory, and based on relatively limited patient cohorts. Prospective validation studies are still required to determine the predictive value of CD73-related biomarkers and to establish standardized criteria for patient stratification in future combination therapies targeting the adenosine pathway.

### 6.3. CD73-Targeted Therapeutic Strategies

Given the central role of the ectonucleotidase CD73 in generating immunosuppressive adenosine within the TME, several therapeutic strategies have been developed to block this metabolic pathway and restore antitumor immunity. These approaches include anti-CD73 monoclonal antibodies, small-molecule inhibitors of CD73 enzymatic activity, and combinatorial strategies with ICIs, particularly those targeting the PD-1/PD-L1 axis. Several CD73-targeting agents and adenosine pathway inhibitors are currently under preclinical and clinical investigation in melanoma and other solid tumors. Their principal mechanisms of action, combination strategies, and current stages of development are summarized in Table 1.

Anti-CD73 monoclonal antibodies are the most clinically advanced strategy for blocking this ectonucleotidase. By blocking CD73 enzymatic activity at the cell surface, they reduce the conversion of AMP into adenosine and attenuate adenosine receptor-mediated immunosuppressive signaling in the TME [82,122,131,132]. Among these, oleclumab (MEDI9447) has demonstrated an acceptable safety profile and pharmacodynamic modulation of the adenosine pathways with PD-1/PD-L1 inhibitors [126,127]. Notably, in a Phase II clinical study (COAST), combining durvalumab (anti-PD-L1) with oleclumab (a CD73 inhibitor) in patients with unresectable stage III non-small-cell lung cancer resulted in higher objective response rates than durvalumab alone. The combination also improved progression-free survival, suggesting steadier disease control over time [133]. Preliminary results of BMS-986179 (NCT02754141) combined with nivolumab in advanced solid tumors, including melanoma, have shown tolerability and modulation of immune markers in the tumor microenvironment, supporting continued investigation [120,134].

Small-molecule inhibitors such as quemliclustat (AB680) offer complementary advantages over antibody-based approaches, including broader tissue diffusion and the ability to inhibit both membrane-associated and soluble forms of CD73 [131]. Preclinical data demonstrate that AB680 reduced adenosine levels in the TME, increasing CD8^+^ T-cell infiltration and activation [14,82]. Consistently, in experimental models, combining AB680 with PD-1 axis blockade led to significantly better tumor control than using PD-1 blockade alone, indicating that CD73 inhibition increases the effectiveness of checkpoint inhibitors-based immunotherapy [14].

Based on these preclinical data, AB680 has advanced to early-phase clinical evaluation, primarily in combination with immunotherapy, to characterize its safety, pharmacodynamics, and immunological impact in patients with advanced solid tumors [128]. An initial Phase I study in healthy volunteers (NCT03677973) demonstrated that AB680 has an acceptable safety and tolerability profile, with pharmacodynamic evidence of CD73 inhibition. Currently, the phase 1b/2 ARC-8 trial is evaluating quemliclustat/AB680 in combination with the anti-PD-1 antibody zimberelimab and standard chemotherapy for the treatment of metastatic pancreatic adenocarcinoma. These studies aim to validate CD73 inhibition as a strategy to potentiate the efficacy of immunotherapy within the TME [14,126].

Beyond CD73-targeted approaches, alternative strategies aimed at reducing downstream adenosine signaling have also been investigated through pharmacological antagonism of the A2A receptor. Preclinical studies demonstrated that A2AR blockade restores T-cell and NK-cell effector functions and enhances responsiveness to PD-1 blockade in murine tumor models [135,136]. Moreover, selective A2AR antagonists, such as istradefylline, have shown immunomodulatory and antitumoral effects, including reduction in malignancy-associated factors and cytotoxicity toward B16-F10 melanoma cells [125] while also suppressing AKT/mTOR signaling and shifting the immune profile toward a pro-inflammatory state in melanoma-bearing mice [137].

From a critical perspective, however, important mechanistic and translation differences exist between A2AR antagonists, such as IST, and anti-CD73 antibodies, such as oleclumab. A2AR antagonists primarily act downstream of adenosine production by selectively blocking receptor-mediated immunosuppressive signaling in effector immune cells. This strategy may restore T-cell and NK-cell activity [135,136], and IST has an already established clinical safety profile in non-oncological indications, such as Parkinson’s disease [125]. However, adenosine accumulation within the TME may persist and continue activating alternative adenosine receptors, and the evidence supporting their use in melanoma remains largely preclinical [137].

In contrast, anti-CD73 antibodies act upstream by limiting adenosine generation within TME, thereby indirectly modulating multiple receptor-mediated immunosuppressive pathways. In addition, oleclumab has already demonstrated clinical feasibility and preliminary efficacy in combination with PD-L1 blockade in solid tumors [126,133], and its investigation in melanoma is more advanced than A2AR antagonists. Therefore, based on currently available evidence, anti-CD73, particularly in combination with ICIs, appears to represent a more clinically advanced and potentially broader strategy for overcoming adenosine-mediated resistance mechanisms in melanoma.

Although CD73 is a promising therapeutic target in CM, several factors may limit the efficacy of its inhibition. A primary example is the functional redundancy of the adenosine-generating machinery within the TME, which can sustain immunosuppressive signaling even in the absence of CD73 [138]. The CD39/CD73 axis is the principal route of adenosine production; however, additional pathways—including the CD38/CD203a axis and alkaline phosphatase (ALP)—can generate adenosine independently of it [83,138]. Consequently, adenosine may continue to accumulate within the TME even after CD73 blockade. Redundancy also operates at the receptor level, since even when CD73 inhibition reduces adenosine availability, the residual adenosine may still engage the low-affinity A2B receptor under hypoxic conditions, thereby sustaining immunosuppressive signaling [88,89]. Moreover, the pathway exhibits intrinsic adaptive capacity, as illustrated by the compensatory upregulation of A2AR and CD73 following PD-1 blockade [121].

The marked heterogeneity of CD73 expression poses an additional obstacle. CD73 is expressed by tumor cells, CAFs, endothelial cells, Tregs, and myeloid populations, and its contribution to adenosine generation varies considerably between tumors and even within distinct regions of the same lesion [82,90,93,94]. This variability may account for the context-dependent prognostic value of CD73, which is unfavorable in most malignancies but favorable in selected contexts, as discussed previously [91,92]. Furthermore, CD73 expression is dynamic rather than static, being modulated by hypoxia, IFN-γ, and TGF-β and further induced by PD-1 blockade, thereby representing a moving therapeutic target [121]. The methods used to quantify CD73, such as immunohistochemistry, soluble CD73, and enzymatic-activity assays, also differ considerably and may yield discordant results, complicating both standardized assessment and patient selection. Finally, adenosine represents only one node within a broader immunosuppressive network that also includes Tregs, IDO, TGF-β, and checkpoints such as LAG-3 and TIM-3 [110]. Given that much of the supporting evidence still derives from poorly immunogenic models such as B16-F10 and from early-phase trials, CD73 inhibition is unlikely to succeed as a single-agent strategy, reinforcing the case for combination strategies and biomarker-guided patient selection.

### 6.4. Evidence of Functional Synergy Between CD73 and PD-1 Blockade

Building on the mechanistic convergence described in Section 5, recent preclinical and translational studies have provided direct experimental evidence that combined CD73 and PD-1 blockade produces effects that exceed those of their strategy alone.

Recent preclinical studies in immunocompetent murine models consistently show that combining CD73 blockade with anti-PD-1 promotes both quantitative and functional expansion of intratumoral CD8^+^ T lymphocytes. This effect is associated with enhanced secretion of IFN-γ, TNF-α, and granzyme B, as well as a coordinated reduction in transcriptional profiles associated with cellular exhaustion, including PD-1, TIM-3, and LAG-3. Notably, these synergistic effects are not fully observed with either monotherapy, suggesting that the adenosine pathway acts as a metabolic barrier that limits the efficacy of checkpoint blockade when used in isolation [82,91].

From a mechanistic perspective, recent evidence indicates that adenosine signaling via A2A and A2B receptors interferes with mTOR- and NFAT-dependent intracellular pathways central to T-lymphocyte activation. This effect compromises signal restoration by PD-1 blockade. CD73 inhibition is suggested to reverse this restrictive metabolic state, enabling TCR reactivation by anti-PD-1 therapy to produce more robust effector responses within the TME [82,89].

Translational data reinforce this model’s clinical relevance. Recent analyses of tumor and circulating samples from patients treated with anti-PD-1 therapy show that elevated adenosine pathway activity is linked to immunologic resistance characterized by low cytotoxicity and predominance of immunosuppressive functions. Integrative transcriptomic and functional data suggest that CD73 inhibition may reprogram this refractory state, potentially converting nonresponsive tumors into those sensitive to immunotherapy [16,94].

Current clinical evidence is primarily from early-phase studies. However, recent trials combining anti-CD73 therapy with PD-1 blockade in advanced solid tumors specifically demonstrated three main findings: consistent modulation of the adenosine pathway, increased infiltration of CD8^+^ T lymphocytes, and a more inflammatory TME. These preliminary biological signals support potential synergy between these therapies [127,139,140].

Additionally, blockade of the CD73/adenosine axis represents a promising strategy to enhance adoptive cellular therapies, such as CAR-T cells, in solid tumors. Adenosine accumulation within the tumor microenvironment (TME) suppresses the effector function of CAR-T cells through activation of the A2A receptor, thereby limiting their clinical efficacy. In this context, interventions aimed at reducing adenosine production via CD73 inhibition may attenuate this immunometabolic suppression, creating a microenvironment more permissive to the cytotoxic activity of these cells and expanding the therapeutic potential of cellular immunotherapies in melanoma and other solid tumors [141].

Collectively, these observations support a model in which CD73 inhibition not only removes an additional immunosuppressive mechanism but also enhances the efficacy of PD-1 blockade, reinforcing the need for the clinical development of combinatorial strategies for the treatment of CM.

## 7. Conclusions and Future Perspectives

The high immunogenicity of CM contrasts with the significant frequency of primary or acquired resistance to PD-1 blockade, underscoring that the isolated removal of signaling checkpoints is insufficient to sustainably restore the antitumor function of T lymphocytes. The evidence discussed indicates that immunosuppression within the TME is not mediated by a single regulatory axis but rather results from the integrated action of metabolic mechanisms and signaling pathways that mutually reinforce one another.

As discussed, the PD-1 and CD73/adenosine axes converge to restrict T-lymphocyte activation and cytotoxic function through distinct but reinforcing mechanisms, collectively sustaining a state of functional exhaustion that persists even in the presence of antigen and isolated checkpoint blockade. The experimental and translational evidence reviewed further demonstrates that this convergence is not merely conceptual: dual blockade produces synergistic immune activation that neither strategy achieves alone, with more CD8^+^ T-cell infiltration, effector cytokine production and reduction in exhaustion markers.

From this perspective, PD-1 blockade restores lymphocyte activation signals, while CD73 inhibition reduces the metabolic barrier caused by adenosine accumulation. Together, this combination creates conditions for more effective and sustained cytotoxic responses by targeting distinct yet integrated aspects of tumor immunosuppression.

However, translating these insights into clinical practice remains challenging. The functional heterogeneity of CD73 within the TME, combined with the lack of validated prospective biomarkers, currently limits accurate patient stratification and the definition of its prognostic and predictive value. In addition, emerging evidence indicates that compensatory immunosuppressive pathways, such as CD39 upregulation and the reprogramming of myeloid cell populations, may further constrain the durability of therapeutic responses to combined strategies.

From a translational standpoint, the successful clinical implementation of combined PD-1 and CD73 blockade will depend on the development of robust stratification approaches capable of identifying patients most likely to benefit. In this context, CD73 expression in tumor tissue and circulating sCD73 activity have been proposed as potential predictive biomarkers of response to immune checkpoint inhibition. Likewise, the proportion of CD8^+^CD73^+^ T lymphocytes and other circulating immunosuppressive subsets may provide additional insight into systemic immunometabolic suppression and improve patient selection.

Biologically, tumors with high adenosine pathway activity, elevated CD73 expression, or enrichment of exhausted T-cell phenotypes may represent a distinct subgroup more likely to respond to dual PD-1/CD73 targeting. In contrast, patients with low CD73 activity or immune-desert tumor microenvironments are less likely to derive meaningful benefit from combination approaches and may require alternative immunotherapeutic strategies. Therefore, integrating immunologic parameters (e.g., tumor-infiltrating lymphocyte density and PD-1 expression), metabolic features (adenosine/CD73 axis activity), and circulating biomarkers may enable a more precise, multidimensional stratification model, ultimately optimizing patient selection and enhancing the clinical efficacy of combination immunotherapy in cutaneous melanoma.

Therefore, although CD73 emerges as a relevant therapeutic target to potentiate the effects of anti-PD-1 immunotherapy in CM, several critical questions remain to be addressed before its incorporation into routine clinical practice. Future investigations should prioritize prospective biomarker-driven clinical trials capable of validating tumor CD73 expression, circulating sCD73 activity, and immune-cell phenotypes as predictive indicators of therapeutic response. In parallel, longitudinal studies evaluating the temporal dynamics of the adenosine pathway during treatment may help define optimal therapeutic timing, mechanisms of acquired resistance, and strategies for treatment sequencing.

Additionally, emerging evidence supports the investigation of multidimensional combination approaches involving PD-1 blockade together with CD73 inhibition and complementary purinergic targets, such as CD39 or A2AR, particularly in patients refractory to current immune checkpoint inhibitors. Furthermore, CD73-targeted therapeutic strategies may in the future enhance the efficacy of adoptive cellular therapies, including CAR-T-cell-based approaches in solid tumors. Advanced translational methodologies may further clarify the cellular interactions governing adenosine-mediated immunosuppression within the melanoma TME and support the development of precision immunotherapy strategies.

Accordingly, integrating immunologic and metabolic biomarkers into clinically applicable stratification models may represent a critical step toward overcoming therapeutic resistance and optimizing individualized immunotherapeutic responses in cutaneous melanoma.

## Figures and Tables

**Table 1:** CD73-targeting agents and adenosine pathway inhibitors in melanoma and other solid tumors: mechanisms, combination strategies, and development status.

Agent	Mechanism of Action	Combination Strategy	Current Development Status
Anti-CD73 Monoclonal antibodies			
Oleclumab (MEDI9447)	Blocks CD73 enzymatic activity and reduces extracellular adenosine production in the TME	Anti-PD-1/anti-PD-L1 combinations	Phase I/II clinical trials in advanced solid tumors
BMS-986179	Inhibits CD73-mediated AMP-to-adenosine conversion	Nivolumab	Early-phase clinical trials
Small-molecule CD73 inhibitors			
Quemliclustat (AB680)	Potent and selective inhibition of CD73 catalytic activity, reducing extracellular adenosine accumulation	PD-1 blockade and chemotherapy	Early-phase clinical trials (including ARC-8)
A2A receptor antagonists			
Istradefylline (IST)	Blocks adenosine-mediated immunosuppressive signaling downstream of CD73	Chemotherapy and immunotherapy (experimental)	Preclinical melanoma studies
Ciforadenant	Inhibits A2A receptor signaling and restores T-cell activity	Anti-PD-1/PD-L1 therapies	Early clinical evaluation in solid tumors
Experimental strategy			
CD73 Genetic Deletion/Experimental Blockade	Prevents adenosine generation in the TME	Immune checkpoint blockade	Preclinical murine melanoma models

## Data Availability

No new data were created or analyzed in this study. Data sharing is not applicable.

## Abbreviations

The following abbreviations are used in this manuscript:

A2AR	Adenosine A2A Receptor
AKT	Protein Kinase A
ADP	Adenosine Diphosphate
ALP	Alkaline Phosphatase
AMP	Adenosine Monophosphate
ATP	Adenosine Triphosphate
B2M	Beta-2 Microglobulin
CAF	Cancer-Associated Fibroblast
CD39	Ecto-Nucleoside Triphosphate Diphosphohydrolase 1
CD73	Ecto-5′-nucleotidase
cAMP	Cyclic Adenosine Monophosphate
CM	Cutaneous Melanoma
CTLA-4	Cytotoxic T-Lymphocyte-Associated Protein 4
DC	Dendritic Cell
ECM	Extracellular Matrix
EMT	Epithelial–Mesenchymal Transition
GPI	Glycosylphosphatididylinositol
HIF-1α	Hypoxia-Inducible Factor 1-Alpha
ICI	Immune Checkpoint Inhibitor
IDO	Indoleamine 2,3-Dioxygenase
IFN-γ	Interferon-Gamma
IL-2	Interleukin-2
IL-10	Interleukin-10
IPRES	Innate Anti-PD-1 Resistance
IST	Istradefylline
JAK 1/2	Janus Kinases 1 and 2
LAG-3	Lymphocyte Activation Gene 3
MDSC	Myeloid-Derived Suppressor Cell
MHC	Major Histocompatibility Complex
mTOR	Mechanism Target Of Rapamycin
NFAT	Nuclear Factor of Activated T-Cells
NK	Natural Killer Cells
OS	Overall Survival
PD-1	Programmed Cell Death Protein 1
PD-L1	Programmed Death Ligand 1
PFS	Progression-Free Survival
P13K	Phosphoinositide 3-Kinase
PKA	Protein Kinase A
Rap1	Ras-Associated protein 1
sCD73	Soluble CD73
TAM	Tumor-Associated Macrophage
TCR	T-Cell Receptor
Tex	T-Cell Exhausted
TGF-β	Transforming Growth Factor Beta
TIM-3	T-Cell Immunoglobulin and Mucin Domain-Containing 3
TILs	Tumor-Infiltrating Lymphocytes
TMB	Tumor Mutational Burden
TME	Tumor Microenvironment
TNF-α	Tumor Necrosis Factor Alpha
Tregs	Regulatory T Cells
UV	Ultraviolet
